# Cross-talk between Two Nucleotide-signaling Pathways in Staphylococcus aureus*[Fn FN2]

**DOI:** 10.1074/jbc.M114.598300

**Published:** 2015-01-09

**Authors:** Rebecca M. Corrigan, Lisa Bowman, Alexandra R. Willis, Volkhard Kaever, Angelika Gründling

**Affiliations:** From the ‡ Section of Microbiology and Medical Research Council Centre for Molecular Bacteriology and Infection, Imperial College London, London SW7 2AZ, United Kingdom and; the § Research Core Unit Metabolomics, Hannover Medical School, Hannover D-306625, Germany

**Keywords:** Bacterial Signal Transduction, Cyclic Nucleotide, Gene Regulation, Microarray, Phosphodiesterases, staphylococcus aureus (S. aureus), Stress Response

## Abstract

Nucleotide-signaling pathways are found in all kingdoms of life and are utilized to coordinate a rapid response to external stimuli. The stringent response alarmones guanosine tetra- (ppGpp) and pentaphosphate (pppGpp) control a global response allowing cells to adapt to starvation conditions such as amino acid depletion. One more recently discovered signaling nucleotide is the secondary messenger cyclic diadenosine monophosphate (c-di-AMP). Here, we demonstrate that this signaling nucleotide is essential for the growth of *Staphylococcus aureus,* and its increased production during late growth phases indicates that c-di-AMP controls processes that are important for the survival of cells in stationary phase. By examining the transcriptional profile of cells with high levels of c-di-AMP, we reveal a significant overlap with a stringent response transcription signature. Examination of the intracellular nucleotide levels under stress conditions provides further evidence that high levels of c-di-AMP lead to an activation of the stringent response through a RelA/SpoT homologue (RSH) enzyme-dependent increase in the (p)ppGpp levels. This activation is shown to be indirect as c-di-AMP does not interact directly with the RSH protein. Our data extend this interconnection further by showing that the *S. aureus* c-di-AMP phosphodiesterase enzyme GdpP is inhibited in a dose-dependent manner by ppGpp, which itself is not a substrate for this enzyme. Altogether, these findings add a new layer of complexity to our understanding of nucleotide signaling in bacteria as they highlight intricate interconnections between different nucleotide-signaling networks.

## Teaser

**Background:** Nucleotide-signaling pathways are complex systems that allow bacteria to rapidly respond to stress.

**Results:** Cyclic diadenosine monophosphate is essential for the growth of *S. aureus*, and its signaling network is intricately interconnected with the stringent response.

**Conclusion:** Cross-talk between different nucleotide-signaling networks is more common than previously anticipated.

**Significance:** Bacteria have evolved intricate signaling networks to survive.

## Introduction

When faced with unfavorable environmental conditions, bacteria employ a variety of small nucleotide-signaling molecules that allow them to rapidly alter cellular physiology to promote survival. Under nutrient limiting conditions, the signaling nucleotide cyclic adenosine monophosphate (cAMP) controls the acquisition of alternative sugar sources (1), whereas the stringent response alarmones guanosine tetra-(ppGpp)^3^ and pentaphosphate (pppGpp) control a transcriptional response allowing cells to adapt during stresses such as amino acid deprivation, carbon source starvation, fatty acid depletion, or osmotic stress (2–4). The stringent response is best characterized in Gram-negative bacteria, where (p)ppGpp is synthesized by the monofunctional enzyme RelA or the bifunctional enzyme SpoT, which also contains hydrolase activity (5). These nucleotides are then responsible for controlling a cellular switch resulting in the down-regulation of pathways involved in active growth and the up-regulation of genes involved in stress adaptation (6). For numerous bacterial species, it has been reported that (p)ppGpp is vital for controlling the transition of bacteria into stationary phase, biofilm formation, sporulation, virulence, antibiotic tolerance, and more recently bacterial persistence (7–10). This is also the case for the Gram-positive bacterial pathogen *Staphylococcus aureus*, where high levels of (p)ppGpp have been shown to affect the virulence of this organism by promoting the formation of persistent and chronic infections (11–14). In *S. aureus*, as well as other Gram-positive species, (p)ppGpp is synthesized in response to amino acid deprivation by RSH, a bifunctional RelA/SpoT homologue (RSH) that contains both a synthase and a hydrolase domain (15, 16). *S. aureus* also produces two other synthases, RelP and RelQ, both of which are monofunctional and transcription of the corresponding genes increases when cells are exposed to cell wall-targeting antimicrobials (17).

A more recently discovered signaling nucleotide is the secondary messenger cyclic diadenosine monophosphate (c-di-AMP), which is predominantly produced by Gram-positive bacteria (18–20). c-di-AMP is synthesized by diadenylate cyclase enzymes and degraded by DHH/DHHA1 domain-containing phosphodiesterases (PDE) (19). In *S. aureus,* the membrane-anchored cyclase DacA produces c-di-AMP. This enzyme is co-transcribed with genes encoding YbbR, a protein that has been implicated in *Bacillus subtilis* in regulating the activity of the DacA homologue CdaA (21), and GlmM, a phosphoglucosamine mutase, which is essential for the production of peptidoglycan precursor glucosamine 6-phosphate. The membrane-anchored PDE GdpP (GGDEF domain protein containing phosphodiesterase) functions to hydrolyze the dinucleotide (22). Under standard laboratory growth conditions, a cytoplasmic c-di-AMP concentration of 2–3 μm has been measured in both the community-acquired methicillin-resistant *S. aureus* strain LAC* and the methicillin-susceptible *S. aureus* laboratory strain SEJ1 (22). For diverse Gram-positive bacteria, it has been shown that high levels of c-di-AMP, due to mutations in *gdpP,* leads to increased resistance toward cell wall-targeting antimicrobials (21–26), acid stress (23, 27, 28), and heat stress (29). Conversely, *S. aureus* strains with increased c-di-AMP levels are more sensitive to osmotic stress, and experiments on *Streptococcus pneumoniae* have highlighted a defect in potassium uptake in mutants with higher levels of this dinucleotide (30, 31). These observations are in line with the recent discoveries that a number of ion transporters are either direct receptors for c-di-AMP (30, 31) or are controlled on a transcriptional level by a c-di-AMP-regulated riboswitch (32). However, little is currently known about the signals and environmental conditions that lead to alterations in cellular c-di-AMP levels, how changes in nucleotide levels alter the cell physiology on a global level, or how the c-di-AMP signaling network is integrated with other cellular responses.

Here, we demonstrate that c-di-AMP is essential for the growth of *S. aureus* and show that its production is elevated in the post-exponential growth phase, indicating that this signaling nucleotide is important for the survival of cells in the late growth phases. By examining the transcriptional profile of cells with high levels of c-di-AMP, a significant overlap with the gene expression pattern observed upon induction of the stringent response is uncovered. Expanding on this, we show that under stress conditions high levels of c-di-AMP result in an RSH-dependent increase in (p)ppGpp production, without c-di-AMP directly binding to the RSH enzyme. Additionally, data are presented showing that (p)ppGpp can also influence c-di-AMP production, highlighting that these nucleotide-signaling networks are interconnected at multiple points.

## EXPERIMENTAL PROCEDURES

### 

#### 

##### Bacterial Strains and Culture Conditions

*Escherichia coli* strains were grown in Luria Bertani broth (LB) and *S. aureus* strains in tryptic soya broth (TSB) or low phosphate chemically defined medium (LP-CDM) at 37 °C with aeration. The LP-CDM was prepared as referenced (33), with the following modifications: the concentration of KH_2_PO_4_ was reduced to 0.4 mm; Gly 50 mg/liter; l-Ser 30 mg/liter; l-Asp 90 mg/liter; l-Lys 50 mg/liter; l-Ala 60 mg/liter; l-Trp 10 mg/liter; l-Met 10 mg/liter; l-His 20 mg/liter; l-Ile 30 mg/liter; l-Tyr 50 mg/liter, and thymine 20 mg/liter was added and 1 m MOPS was used to buffer the media to pH 7.2. When required, media were supplemented with antibiotics and inducers as indicated in Table 1.

**TABLE 1:** **Bacterial strains used in this study** Antibiotics were used at the following concentrations: for *E. coli* cultures: kanamycin (KanR) 30 μg/ml, ampicillin (AmpR) 100 μg/ml, gentamicin (GnR) 20 μg/ml, and chloramphenicol (CamR) 10 μg/ml; for *S. aureus* cultures: erythromycin (ErmR) 10 μg/ml, kanamycin (KanR) 90 μg/ml, and chloramphenicol (CamR) 7.5 μg/ml. Inducers were used at the following concentrations: isopropyl 1-thio-β-d-galactopyranoside 1 mm; Atet 50 ng/ml. nt means nucleotide.

Strain	Relevant features	Ref.
***Escherichia coli* strains**		
XL1-Blue	Cloning strain, TetR ANG127	Stratagene
DH5α	Cloning strain-ANG397	56
ANG203	pCN49 in *E. coli*: AmpR	34
ANG292	pCL55iTETr862 in XL1-Blue: AmpR	31
ANG1429	pKOR1 in *E. coli* strain DB3.1: AmpR	36
ANG1632	pCN34iTET in *E. coli*: AmpR	22
ANG1676	pCN38 in XL1-Blue: AmpR	34
ANG1861	pET28b-*gdpP*_84–655_ in BL21 (DE3): KanR	22
ANG1970	pET28b-*disA* in BL21 (DE3): KanR	22
ANG1971	pET28b-*dacA* in BL21 (DE3): KanR	22
ANG2246	pCN49iTET in XL1-Blue: AmpR	This study
ANG2247	pCN49iTET-*dacA* in XL1-Blue: AmpR	This study
ANG2250	pKOR1-*dacA*::*kan* in DH5α: AmpR	This study
ANG2352	pREP4 in XL1-Blue: KanR	Invitrogen
ANG2547	pCL55iTETr862-*dacA* in XL1-Blue pREP4: AmpR, KanR	This study
ANG2815	pVL847 in XL1-Blue: AmpR	57
ANG2882	pVL847-*ktrA* in XL1-Blue: AmpR	This study
ANG2883	pVL847-*ktrA* in BL21 (DE3): AmpR	This study
ANG2890	pVL847 in BL21 (DE3): AmpR	This study
ANG3030	pET28b-*relA* in XL1-Blue: KanR	This study
ANG3031	pET28b-*relA* in BL21 (DE3): KanR	This study
ANG3032	pET28b-*gppA* in XL1-Blue: KanR	This study
ANG3033	pET28b-*gppA* in BL21 (DE3): KanR	This study
ANG3162	pKOR1-*rsh*_syn_ in DH5α: AmpR	This study
ANG3189	pVL847-*rsh* in T7IQ: GnR, CamR	Lab strain collection

***Staphylococcus aureus* strains**		
SEJ1	RN4220*spa*; protein A negative derivative of RN4220; ANG314	58
ANG467	NMΔΦ4; Newman strain with deletion of phage NM4, which allows integration of pCL55-derived plasmids	31
LAC*	Erm sensitive CA-MRSA LAC strain (AH1263); ANG1575	59
SH1000	Strain 8325–4 with repaired defect in *rsbU*	60
ANG1961	LAC*gdpP::*kan*: KanR	22
ANG2253	SEJ1 *dacA*::*kan* pCN49iTET-*dacA*: KanR, ErmR	This study
ANG2542	LAC* pCL55iTETr862-*dacA*: CamR	This study
ANG2543	LAC*dacA::*kan* pCL55iTETr862-*dacA* (LAC*i*dacA*): KanR, CamR	This study
ANG2545	SEJ1 pCL55iTETr862-*dacA*: CamR	This study
ANG3163	SEJ1 pKOR1-*rsh*_syn_: CamR	This study
ANG3165	LAC*rsh_syn_	This study
ANG3188	SH1000 Δ*ahpC* Δ*katA*	61
ANG3306	LAC* Δ*gdpP rsh*_syn_	This study
HG001	NCTC8325 *rsbU* repaired ANG3401	62
HG001–230	HG001 *relQ*_syn_: deleted for nt 343–429; ANG3402	17
HG001–229	HG001 *relP*_syn_: deleted for nt 450–536; ANG3403	17
ANG3407	HG001 Δ*gdpP*	This study
ANG3408	HG001 Δ*gdpP relP*_syn_	This study
ANG3409	HG001 Δ*gdpP relQ*_syn_	This study

##### Plasmid and Strain Construction

Strains used in this study are listed in Table 1 and primers used are listed in Table 2. Plasmid pCL55iTETr862-*dacA* for anhydrotetracycline (Atet)-inducible *dacA* expression in *S. aureus* was created by amplifying the *dacA* gene using primers ANG1450/ANG1451 from *S. aureus* SEJ1 chromosomal DNA. The resulting PCR product was digested with AvrII and SacII and cloned into pCL55iTETr862 (22) that had been digested with the same enzymes. pCN49iTET was constructed by isolating the erythromycin (Erm) cassette from pCN49 (34) following digestion with AvrII and SacII and cloning it into pCN34iTET (22) that had been cut with the same enzymes. This resulted in the replacement of the kanamycin cassette in pCN34iTET with the Erm marker. Plasmid pCN49iTET-*dacA* was constructed by amplifying the *dacA* gene from *S. aureus* SEJ1 genomic DNA with primers ANG928/ANG929. The PCR product was digested with KpnI and EcoRI and ligated with pCN49iTET that had been cut with the same enzymes. Plasmid pET28b-*relA* was produced by amplifying the *relA* gene from *E. coli* MG1655 chromosomal DNA using primers ANG1704/ANG1705. The resulting PCR product was digested with NdeI and EcoRI and cloned into pET28b that had been digested with the same enzymes. pET28b-*gppA* was created by amplifying the *gppA* gene from *E. coli* MG1655 chromosomal DNA using primers ANG1721/ANG1722 and inserting the NcoI- and HindIII-cut PCR product into NcoI- and HindIII-digested pET28b. Plasmid pVL847-*ktrA* was produced by digesting plasmid pET28b-*ktrA* (31) with NdeI and HindIII and cloning the resultant product into pVL847 that had been digested with the same enzymes. All plasmids were initially transformed into *E. coli* strain XL1-Blue, and sequences of all inserts were verified by fluorescence-automated sequencing at the Medical Research Council Clinical Science Centre Sequencing Facility at Imperial College London. pET28b- and pVL847-derived plasmids were subsequently transformed into BL21(DE3) for protein induction. The *S. aureus* expression vector pCL55iTETr862-*dacA* was initially electroporated into SEJ1, where it integrates into the *geh* locus, before being phage-transduced into LAC* using Φ85 to create strains ANG2545 and ANG2542, respectively.

**TABLE 2:** **Primers used in this study** Restriction sites in primer sequences are underlined.

No.	Name	Sequence
ANG928	F-KpnI-dacA	TTTGGTACCTATTACCCGGAGGAGATG
ANG929	R-EcoRI-dacA	CCCGAATTCCATATTATTTCACACCTT
ANG946	F-AttB1	GGGGACAAGTTTGTACAAAAAAGCAGGCTTC
ANG947	R-AttB2	GGGGACCACTTTGTACAAGAAAGCTGGGTC
ANG1191	R3′up-5′Kan-dacA	CATTTTAGCCATAACATCTCCTCCGGGTAATATTTT
ANG1192	F5′Kan-3′up-dacA	GGAGGAGATGTTATGGCTAAAATGAGAATATCACCG
ANG1193	R3′Kan-5′down-dacA	CAAGCAACTCTTCTAAAACAATTCATCCAGTAAAAT
ANG1194	F5′down-3′Kan-dacA	GAATTGTTTTAGAAGAGTTGCTTGCTGAACATTGGT
ANG1195	R-attB2-dacA	ACAAGAAAGCTGGGTCCATTTATATAAGCCTTCGTTTCACTTGGTTG
ANG1196	F-dacA-check	GTGTAGCTATTGCTTGATAATGG
ANG1197	R-dacA-check	CCTAATTTAAATGCCAATTCAGGTG
ANG1218	F-attB1-dacA	ACAAAAAAGCAGGCTTCGAGGTTTCAGCATCAATTGAAAATAACAG
ANG1450	F-AvrII-dacA	GGGCCTAGGTATTACCCGGAGGAGATG
ANG1451	R-SacII-dacA	GGGCCGCGGCATATTATTTCACACCTT
ANG1704	F-NdeI-relA	GGGCATATGGTTGCGGTAAGAAGTGCACATATC
ANG1705	R-EcoRI-relA	AAAGAATTCCTAACTCCCGTGCAACCGACGC
ANG1707	F-attB-RSH	ACAAAAAAGCAGGCTTCATTAGTTGAAAAATTAGGCGGTATCGTAGT
ANG1708	R-attB-RSH	ACAAGAAAGCTGGGTCCAAATCCTACAGCTGCGAATAAATCATCTT
ANG1709	R-YQS-RSH	TGTAGTATGCAACAAATTTTGTTTAGGCATTGCAAT
ANG1710	F-YQS-RSH	AAACAAAATTTGTTGCATACTACAGTAGTAGGCCCA
ANG1721	F-NcoI-gppA	GGGCCATGGGTTCCACCTCGTCGCTG
ANG1722	R-HindIII-gppA	GGGAAGCTTATGCACTTCCAGCGGCCAG
ANG1724	F-RSH-check	CGCAAAAGACAGAGATGTTG
ANG1725	R-RSH-check	CTAGTTCCAAACTCTTGTTAC

For deletion of the *dacA* gene in *S. aureus*, 1-kb fragments up- and downstream of *dacA* were amplified from SEJ1 genomic DNA using primers pairs ANG1218/ANG1191 and ANG1194/ANG1195, which incorporate 5′ and 3′ attB sites, respectively. A kanamycin cassette was amplified from plasmid pCN34iTET using primer pair ANG1192/ANG1193. Purified PCR products were then fused by splice overlap extension PCR using primers ANG946/ANG947. The purified PCR product was recombined with pKOR1 and transformed into *E. coli* strain DH5α. The plasmid was subsequently electroporated into SEJ1 and stably maintained at 30 °C in the presence of 10 μg/ml chloramphenicol (Cam). Shifting the temperature to 43 °C resulted in a single crossover event and insertion of the plasmid into the chromosome. Upon confirmation of chromosomal insertion by PCR, the *dacA* complementation plasmid pCN49iTET-*dacA* was introduced into this strain by electroporation, and transformants were recovered at 37 °C on plates containing 5 μg/ml Cam, 10 μg/ml Erm, and 50 ng/ml Atet. The transformants were subsequently propagated at 30 °C in the absence of Cam, while selecting for and inducing the covering plasmid with 10 μg/ml Erm and 50 ng/ml Atet, which resulted in excision of pKOR1 and deletion of the chromosomal copy of the *dacA* gene creating strain ANG2253. Replacement of the *dacA* gene was confirmed using primer pair ANG1196/ANG1197. Introduction of and selection for pCN38, a plasmid with the same replication of origin as pCN49iTET, was attempted to remove the complementation plasmid pCN49iTET-*dacA* by plating the strain on 10 μg/ml Cam plates; however, this was unsuccessful. Strain LAC* *dacA*::*kan* pCL55iTETr862-*dacA* (LAC*i*dacA*) was created by phage transducing the *dacA*::*kan* deletion into strain ANG2542 in the presence of 50 ng/ml Atet.

An *rsh*_syn_ mutant was constructed as described previously (35). To this end, 1 kb up- and downstream fragments containing overlapping 3′ and 5′ regions, respectively, that lacked nine bases encoding for the three conserved amino acids YQS at position 308–310 in the (p)ppGpp synthesis domain of the enzyme RSH were amplified with primer pairs ANG1707/ANG1709 and ANG1708/ANG1710. Purified PCR products were then fused by splice overlap extension PCR using primers ANG946/ANG947, which also added attB sites for recombination with pKOR1. The resulting PCR product was recombined with pKOR1 and transformed into DH5α resulting in strain ANG3162. pKOR1-*rsh*_syn_ was then electroporated into *S. aureus* SEJ1 and stably maintained at 30 °C in the presence of 10 μg/ml Cam. The plasmid was then isolated and electroporated into LAC*, and transformants were recovered at 30 °C. Allelic exchange was performed as described previously (36) resulting in strain LAC*rsh_syn_ (ANG3165). Replacement of the wild type *rsh* gene in strain ANG3165 with the *rsh*_syn_ variant was confirmed by sequencing. Strains LAC*rsh_syn_Δ*gdpP,* HG001Δ*gdpP,* HG001*relP*_syn_Δ*gdpP,* and HG001*relQ*_syn_Δ*gdpP* were created by phage transducing the *gdpP*::*kan* deletion from strain ANG1961 into the appropriate background strain.

##### Growth Curves and Determination of CFUs

*S. aureus* strains were grown overnight in TSB medium with the appropriate antibiotics and inducers. Overnight cultures of wild type (Newman or LAC*) strains and LAC*Δ*gdpP* strains were diluted in TSB to a starting *A*_600_ of 0.01 and 0.05, respectively. The LAC*i*dacA* strain was washed three times with TSB and diluted to an *A*_600_ of 0.01 in media with and without 50 ng/ml Atet. Cultures were incubated at 37 °C with aeration, and once cells reached an *A*_600_ of 0.5, cultures were diluted in fresh media with the appropriate inducers to an *A*_600_ of 0.01 for wild type and LAC*i*dacA* strains or an *A*_600_ of 0.05 for LAC*Δ*gdpP.* Cultures were grown for 8 h, and *A*_600_ values determined at 2-h intervals. Growth curves were performed in triplicate, and averages and standard deviations are plotted. CFUs per ml of culture of LAC*i*dacA* in the presence and absence of 50 ng/ml Atet were determined by emulsifying a colony in 1 ml of TSB, normalizing the *A*_600_ to 0.05, performing serial dilutions to 10^−4^, and plating 50 μl on the appropriate plates. Plates were then incubated at 37 °C, and colonies were enumerated after overnight growth.

##### Protein Purifications

Proteins were purified from 2 to 4 liters of *E. coli* cultures. Unless otherwise specified, cultures of the respective strains were grown to an *A*_600_ 0.5–0.7; protein expression was induced with 1 mm isopropyl 1-thio-β-d-galactopyranoside, and the cultures were subsequently incubated overnight at 16 °C. RSH protein expression was induced with 1 mm isopropyl 1-thio-β-d-galactopyranoside for 6 h at 30 °C after overnight growth of the culture at 30 °C. DisA, DacA, GdpP, RSH, KtrA, MBP, and GppA proteins were purified by nickel affinity and size exclusion chromatography as described previously (37). The RelA protein was purified by nickel chromatography as described by Jenvert *et al.* (38). Protein concentrations were calculated based on *A*_280_ readings.

##### Western Blotting

Bacteria from 1 or 2 ml of *S. aureus* culture aliquots were collected by centrifugation and subsequently suspended in TSM buffer (50 mm Tris-HCl, pH 7.5, 0.5 m sucrose, 10 mm MgCl_2_) containing 100 μg/ml lysostaphin and 20 μg/ml DNase normalized for *A*_600_ readings of the bacterial cultures, *i.e.* 1-ml samples from a culture with an *A*_600_ of 1 were suspended in 50 μl of buffer. Samples were incubated at 37 °C for 30 min and subsequently mixed 1:1 with 2× SDS protein sample buffer. Aliquots were separated on 7.5% (for the detection of GdpP) or 12% (for the detection of DacA) SDS-polyacrylamide gels, and proteins were subsequently transferred to PVDF membranes. DacA and GdpP were detected using anti-DacA or anti-GdpP antibodies (Covalab) at a 1:10,000 dilution and HRP-conjugated anti-rabbit IgG (Cell Signaling Technologies) at a 1:10,000 dilution as secondary antibody. Blots were developed by enhanced chemiluminescence and imaged using a LAS-3000 Fuji Imager (FUJIFILM). Band intensities were quantified using ImageJ software.

##### Quantification of c-di-AMP by LC-MS/MS

Quantification of intracellular levels of c-di-AMP was performed as described previously (22). The micromolar concentration of c-di-AMP in cells was calculated as follows: CFUs per ml of culture were determined for each time point by removing 25 μl of culture, normalizing the *A*_600_ to 0.05, performing serial dilutions to 10^−4^, and plating 50 μl on the appropriate agar plates. Plates were then incubated at 37 °C, and colonies were enumerated after overnight growth. The nanograms of c-di-AMP/ml culture was divided by the number of CFU/ml to obtain the nanograms of c-di-AMP/CFU. The nanograms of c-di-AMP/CFU was converted to a micromolar concentration based on a previously measured average cell diameter for LAC* cells of 1.188 μm (22), spherical shape of *S. aureus* cells with a volume of 4/3πr^3^, and a molecular weight for c-di-AMP (free acid) of 658.4 g/liter. A LAC*i*dacA* culture grown in the absence of the inducer Atet, as well as cells exposed to 5 mm H_2_O_2_, 2.4 mm HOCl, or 0.05 μg/ml mupirocin contain large numbers of nonviable cells, and hence CFU/ml counts could not be obtained. The results are therefore calculated as nanograms of c-di-AMP/mg bacterial dry weight.

##### Synthesis of Radiolabeled c-di-AMP and (p)ppGpp

^32^P-Labeled c-di-AMP with a concentration of 9.99 nm was synthesized as described previously (31). For use in hydrolysis and inhibition assays, [^32^P]c-di-AMP with a final concentration of 2 mm was synthesized as above but with the addition of 4 mm cold ATP to the reaction mixture (31). ^32^P-Labeled pppGpp was synthesized from [α-^32^P]GTP (PerkinElmer Life Sciences) in 50 mm Tris, pH 8, 15 mm MgOAc, 60 mm KOAc, 30 mm NH_4_OAc, 0.2 mm EDTA, 0.5 mm PMSF, 15% MeOH synthesis buffer by incubating 55.5 nm [α-^32^P]GTP with 1 μm RelA protein, 2 mm ATP, and 2 mm GTP, and reactions were incubated overnight at 30 °C. The reaction was heat-inactivated by incubating for 5 min at 95 °C, and the RelA protein was removed by centrifugation. To synthesize [^32^P]ppGpp, [^32^P]pppGpp was incubated with 1 μm GppA protein for 15 min at room temperature. The reaction was subsequently heat-inactivated, and the GppA protein was removed by centrifugation. Reaction products were visualized by spotting 2 μl on PEI-cellulose F TLC plates (Merck Millipore) and separated in 1.5 m KH_2_PO_4_, pH 3.6, buffer. The radioactive spots were visualized using a FLA 7000 Typhoon PhosphorImager.

##### In Vitro GdpP Phosphodiesterase Activity Assays

The enzymatic activity of purified GdpP protein lacking the N-terminal transmembrane helices was determined against both radiolabeled c-di-AMP and (p)ppGpp. The reactions were set up in 50 mm Tris, pH 8.5, 0.1 mm MnCl_2_, 20 mm KCl buffer with 1 μm recombinant GdpP (rGdpP) protein. The reactions were initiated by the addition of 20 μm radiolabeled c-di-AMP or (p)ppGpp substrates and subsequently incubated at room temperature with samples withdrawn at 0, 5, 10, 30, and 60 min. The reactions were stopped by heat inactivation for 5 min at 95 °C, and the rGdpP was removed by centrifugation. For the ppGpp inhibition assays, the above reactions containing 20 μm [^32^P]c-di-AMP and 1 μm rGdpP were incubated with ppGpp (tebu-bio) ranging from 0 to 5 mm. Samples were incubated at room temperature for 5 min before the reactions were stopped as described above. Two μl of the different samples were spotted on PEI-cellulose F TLC plates (Merck Millipore), and the TLC plates were run in 1:1.5 (v/v) saturated NH_4_SO_4_ and 1.5 m KH_2_PO_4_, pH 3.6, buffer for visualizing c-di-AMP and in 1.5 m KH_2_PO_4_, pH 3.6, buffer for visualizing (p)ppGpp. The radioactive spots were visualized using an FLA 7000 Typhoon PhosphorImager, and data were quantified using ImageQuantTL.

##### Differential Radial Capillary Action of Ligand Assay

This assay was performed as described previously with slight modifications (31, 39). Briefly, *E. coli* whole cell lysates or 40 μm purified protein in 40 mm Tris, pH 7.5, 10 mm MgCl_2_, and 100 mm NaCl with 1 mm cold ATP were mixed with ∼1 nm [^32^P]c-di-AMP and incubated at room temperature for 5 min. Reactions were spotted onto nitrocellulose membrane and air-dried, and the radioactive spots were visualized using a FLA 7000 Typhoon PhosphorImager.

##### Measurement of (p)ppGpp Levels in S. aureus

*S. aureus* strains LAC*, LAC*Δ*gdpP*, LAC*rsh_syn_, LAC*rsh_syn_Δ*gdpP*, HG001, HG001Δ*gdpP*, HG001*relP*_syn_Δ*gdpP*, and HG001*relQ*_syn_Δ*gdpP* were grown overnight in low phosphate CDM at 37 °C. Cultures were diluted to an *A*_600_ of 0.05 and grown until an *A*_600_ of 0.5. The cultures were once again diluted to an *A*_600_ of 0.05 to ensure all cells were in the exponential phase. Five-ml cultures were grown until an *A*_600_ of 0.5 prior to the addition of 3.7 MBq of [^32^P]H_3_PO_4_ to each culture. The cultures were grown for a further 3 h at 37 °C and subsequently normalized for absorbance and split into 2 aliquots. One of these aliquots was supplemented with 60 μg/ml mupirocin, and all cultures were further incubated for 30 min at 37 °C. One-ml culture aliquots were removed and mixed with 200 μl of 2 m formic acid and subsequently subjected to four freeze/thaw cycles. Samples were incubated on ice for 30 min and centrifuged at 17,000 × *g* for 5 min. Ten μl of the supernatant fractions were subsequently spotted on PEI-cellulose F TLC plates (Merck Millipore), nucleotides separated, and TLC plates developed using a 1.5 m KH_2_PO_4_, pH 3.6, buffer. The radioactive spots were visualized using an FLA 7000 Typhoon PhosphorImager, and data were quantified using ImageQuantTL software.

##### Electron Microscopy

*S. aureus* strains were grown overnight in TSB medium with the appropriate antibiotics and inducers. Overnight cultures of LAC* and LAC*Δ*gdpP* strains were diluted to a starting *A*_600_ of 0.01 and 0.05, respectively. The LAC*i*dacA* strain was washed three times with TSB and diluted to an *A*_600_ of 0.01 in media lacking Atet. Cultures were incubated at 37 °C with aeration, and once cells reached an *A*_600_ of 1, the cultures were diluted in fresh media to an *A*_600_ of 0.01 for wild type and LAC*i*dacA* strains and an *A*_600_ of 0.05 for Δ*gdpP.* Cultures were grown for a further 4 h at 37 °C, and aliquots were prepared for EM as described previously (40, 41). Briefly, bacteria from the equivalent of 20 ml of culture with an *A*_600_ of 1 were collected by centrifugation at 8,000 × *g*, washed twice with 10 ml of 0.2 m cacodylate buffer, pH 7.1. Bacteria were suspended in 2 ml of 0.2 m cacodylate buffer, pH 7.1, containing 4% glutaraldehyde and incubated for 1 h at 4 °C. Fixed bacteria were pelleted by centrifugation at 10,000 × *g* for 10 min, washed twice with 1 ml of 0.2 m cacodylate buffer, pH 7.1, and subsequently transferred to poly-l-lysine-coated 35-mm round tissue culture dishes. The cells were overlaid with 1 ml of 0.2 m cacodylate buffer, pH 7.1, containing 4% glutaraldehyde and incubated for 1 h at room temperature. The bacteria were subsequently washed six times with 2 ml of 0.2 m cacodylate buffer, pH 7.1, and subsequently processed for electron microscopy as described previously (40). Images were taken on an FEI Tecnai GZ transmission electron microscope at the Henry Wellcome Trust Imaging Centre, St. Mary's Campus, Imperial College London.

##### RNA Extraction

LAC* and LAC*Δ*gdpP* were grown overnight in TSB. Cultures were diluted to an *A*_600_ of 0.05 and grown until the *A*_600_ reached 1. Cultures were diluted into fresh TSB to an *A*_600_ of 0.05 and grown for 4 h at 37 °C. 10-ml aliquots of cells were mixed with 23.3 ml of GTC buffer (5 m GTC, 0.5% *N*-lauryl sarcosine, 0.1 m β-mercaptoethanol, 0.5% Tween 80, 10 mm Tris, pH 7.5) and centrifuged at 7,000 × *g* for 10 min. The cell pellet was suspended in 1 ml of RNApro (MPBiomedicals, LLC), and cells were lysed in a Fast-Prep machine three times for 45 s at setting 6 (FP120, MPBiomedicals, LLC). Beads were separated by centrifugation at 12,000 × *g* for 5 min at 4 °C. The upper phase was mixed with 300 μl of chloroform and incubated for 5 min at room temperature, and samples were subsequently centrifuged at 12,000 × *g* for 5 min at 4 °C. The upper phase was transferred to a fresh tube and was once again extracted with 300 μl of chloroform as before. Following the second extraction, the upper phase was mixed with 500 μl of 100% ethanol and incubated at −20 °C for 30 min. Nucleic acids were subsequently pelleted by centrifugation at 12,000 × *g* for 5 min at 4 °C, washed with 75% ethanol, suspended in 100 μl of RNAsecure (Ambion), and incubated at 60 °C for 10 min. DNA was removed using the RNeasy RNA cleanup kit (Qiagen), followed by treatment with TURBO DNase (Ambion). One μl of SUPERASEin (Ambion) was subsequently added to every 20 μl of RNA sample, and aliquots were stored at −80 °C. Sample integrity was assessed on a bioanalyzer (Agilent Technologies, Inc.) prior to use in microarray experiments.

##### Microarray Analysis

For each replicate, 2 μg of RNA was labeled with Cy3 dCTP (GE Healthcare) using random primers (Invitrogen) and Superscript II reverse transcriptase (Invitrogen). Fluorescently labeled cDNAs were purified and hybridized onto BμG@S SAv2.1.0 microarrays provided by the Bacterial Microarray Group at St. George's, University of London, using a one-color microarray-based gene expression system. Washing, scanning, and feature extraction procedures were performed as described previously (42). Array design is available in BμG@Sbase (accession number A-BUGS-42) and also ArrayExpress (accession number A-BUGS-42). Statistical analyses were performed using GeneSpring (Agilent Technologies). Differentially expressed genes were defined as those that showed >2-fold up- or down-regulation with respect to the wild type, with a *p* value of <0.05 determined by two-way analysis of variance with Benjamini and Hochberg false discovery rate correction. A total of 465 differentially expressed genes were identified from the raw data, which was reduced to 307 genes once duplicate genes were removed. Fully annotated microarray data have been deposited in BμG@ base (accession number E-BUGS-158) and also ArrayExpress (accession number E-BUGS-158).

## RESULTS

### 

#### 

##### Intracellular c-di-AMP Levels Increase in S. aureus upon Entry into Stationary Phase

**FIGURE 1.**
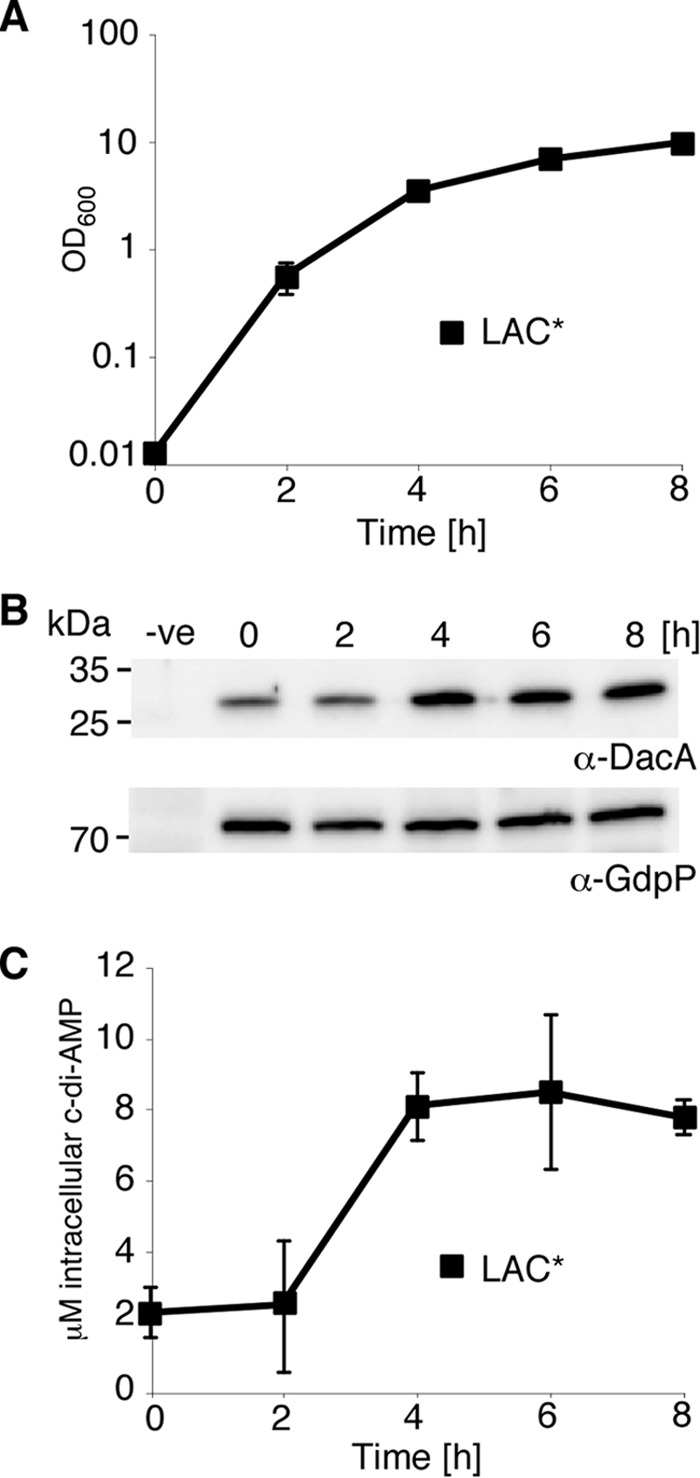
**c-di-AMP levels vary during the growth of *S. aureus*.** *A,* growth curve of *S. aureus* strain LAC*. LAC* was diluted to an *A*_600_ of 0.01 and grown to the exponential phase prior to dilution to an *A*_600_ of 0.01 (time = 0 h), and growth was monitored and recorded for 8 h. The average *A*_600_ values and standard deviations from three independent experiments were plotted. *B,* DacA and GdpP protein production during the growth of *S. aureus* strain LAC*. Samples were taken at the indicated time points from an *S. aureus* LAC* culture; whole cell lysates were prepared, and the DacA and GdpP proteins were detected by Western blot using protein-specific antibodies. Samples prepared from LAC*i*dacA* grown in the absence of Atet for 2 h (see Fig. 2*A*) and LAC*Δ*gdpP* cultures (Fig. 3*A*) were used as negative controls on DacA and GdpP Western blots, respectively. All protein band intensities were quantified using ImageJ. The positions of protein molecular mass markers (in kDa) are indicated on the *left. C,* intracellular c-di-AMP levels. The intracellular concentration of c-di-AMP during the growth of strain LAC* was determined by LC-MS/MS and its micromolar concentration calculated. The average values and standard deviations of three independent experiments are plotted.

In *S. aureus,* c-di-AMP is produced by the cyclase DacA and hydrolyzed by the PDE GdpP. To examine whether production of these enzymes is regulated during bacterial growth, DacA and GdpP protein levels were determined during the growth of the methicillin-resistant *S. aureus* strain LAC* (Fig. 1*A*), as well as the methicillin susceptible strain Newman (data not shown). As revealed by Western blot and densitometric analysis of protein band intensities, GdpP was expressed constitutively throughout growth (Fig. 1*B*, data not shown), whereas expression of the cyclase DacA was increased 2.7 ± 0.7-fold as the cells entered the late exponential to stationary growth phase (Fig. 1*B*, data not shown). As determined by LC-MS/MS analysis of cytoplasmic extracts, the increase in DacA production at the 4-h time point correlated with an increase in c-di-AMP levels. Bacteria in the exponential growth phase (2-h time point) contained 2.43 ± 1.87 μm c-di-AMP, and the concentration increased to 8.09 ± 0.96 μm at the 4-h time point when cells reached the late exponential/early stationary growth phase and remained high in the stationary phase (Fig. 1*C*). These results indicate that the levels of DacA and c-di-AMP are regulated during the growth of *S. aureus* and that the concentration of this signaling molecule is highest in slow growing or nondividing cells.

##### c-di-AMP Is Essential for the Growth of S. aureus

Previous reports have indicated that c-di-AMP is essential for the viability of Gram-positive species, as attempts to delete c-di-AMP synthase genes in *Listeria monocytogenes* (20, 23), *B. subtilis* (21, 24), *Mycoplasma pulmonis* (43), *Mycoplasma genitalium* (44), and *S. pneumoniae* (45) failed. To establish whether *dacA* is also essential in *S. aureus*, we attempted to replace the *dacA* gene with a kanamycin cassette; however, this was only possible in the presence of the complementation plasmid pCN49iTET-*dacA*. Additionally, attempts to move the Δ*dacA*::*kan* chromosomal region by phage transduction into LAC*, or into the more readily genetically manipulatable laboratory strain RN4220, were unsuccessful. The Δ*dacA*::*kan* deletion could be transduced into strain LAC* pCL55iTETr862-*dacA,* which contained a chromosomally integrated plasmid for the expression of *dacA* from a tetracycline-inducible promoter, however only in the presence of the inducer Atet. This created strain LAC*Δ*dacA*::*kan* pCL55iTETr862-*dacA,* from here on out referred to as LAC*idacA.

**FIGURE 2.**
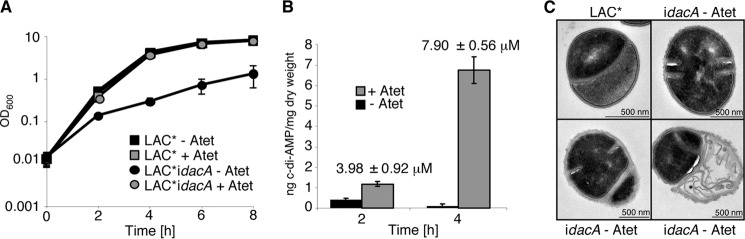
**c-di-AMP depletion negatively affects growth of *S. aureus*.** *A,* growth of *S. aureus* strains LAC* and LAC*i*dacA*. Growth curves of LAC* were performed as described in Fig. 1*A*. An overnight culture of LAC*i*dacA* grown in the presence of 50 ng/ml Atet was washed, diluted to an *A*_600_ of 0.01, and grown to an *A*_600_ of 0.5 in the presence or absence Atet. Cultures were again diluted to an *A*_600_ of 0.01 (time = 0 h) and grown in the presence and absence of 50 ng/ml Atet for 8 h. Growth curves were performed three times, and average *A*_600_ readings and standard deviations are plotted. *B,* intracellular c-di-AMP levels. The intracellular concentration of c-di-AMP in strain LAC*i*dacA* grown in the presence or absence of Atet was determined at the 2- and 4-h time points by LC-MS/MS. As LAC*i*dacA* cultures grown in the absence of Atet contain a large number of nonviable cells, c-di-AMP concentrations are presented as nanograms of c-di-AMP/mg of dry weight. CFU counts were obtained for LAC*i*dacA* grown in the presence of Atet, and hence the micromolar concentration of c-di-AMP is also indicated. Average values and standard deviation of three experiments are plotted. *C,* TEM images of wild type LAC* and LAC*i*dacA* grown in the absence of Atet. Samples were taken at the 4-h time point during bacterial growth and prepared for TEM as described under “Experimental Procedures.” Representative images and *scale bars* are shown.

Strain LAC*idacA allows for conditional *dacA* expression and can now be used to investigate the effects of *dacA* depletion on growth and cell morphology. In the presence of 50 ng/ml Atet, LAC*idacA grew like the wild type LAC* strain and also produced similar amounts of c-di-AMP (Fig. 2, *A* and *B*). In the absence of inducer, the strain showed a significant growth defect (Fig. 2*A*), a 4-log decrease in viable cell count, and as expected drastically reduced c-di-AMP levels (Fig. 2*B*). However, no complete growth arrest was seen even in the absence of inducer, which is likely due to basal transcription from the iTET promoter. To examine the effects of low c-di-AMP levels on cell morphology, samples were analyzed by electron microscopy (EM). In contrast to wild type cells, LAC*idacA cells grown in the absence of Atet contained multiple incomplete septa and in some cases lacked their cytoplasmic content entirely (Fig. 2*C*). Together these data indicate that *dacA* is essential for the growth of *S. aureus* and that cells that produce insufficient amounts of c-di-AMP are unable to progress through the cell division cycle.

**FIGURE 3.**
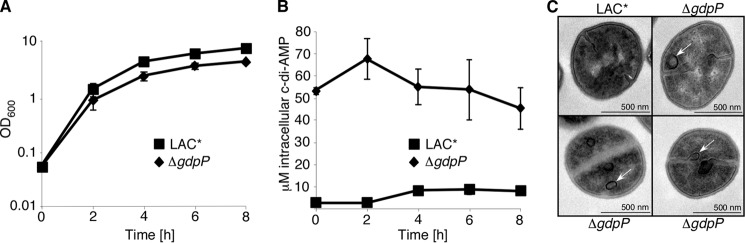
c-di-AMP overproduction in *S. aureus* strain LAC*. *A,* growth of *S. aureus* strains LAC* and LAC*Δ*gdpP*. Cultures were diluted to an *A*_600_ of 0.05 and grown to exponential phase prior to dilution to an *A*_600_ of 0.05 and growth for 8 h. The average *A*_600_ readings and standard deviations of three independent experiments are plotted. *B,* intracellular c-di-AMP levels. The concentration of c-di-AMP in the cytoplasm of LAC* and LAC*Δ*gdpP* was determined by LC-MS/MS and plotted as described in Fig. 1*C. C,* TEM images of wild type LAC* and LAC*Δ*gdpP S. aureus* strains. Samples were taken at the 4-h time point during bacterial growth and prepared for TEM as described under “Experimental Procedures.” Representative images and *scale bars* are shown. *White arrows* indicate extramembranous material visible in the *gdpP* mutant strain.

We have previously reported that strain LAC*Δ*gdpP*, inactivated for the c-di-AMP-specific PDE GdpP, has increased c-di-AMP levels during late growth phases (22). Here, we show that the concentration of c-di-AMP is maintained at a high and relatively constant level throughout all growth phases in this mutant (Fig. 3*B*). Bacteria with high levels of cellular c-di-AMP also have a growth defect, although this is not as severe as when the levels of c-di-AMP are low (Fig. 3*A*). LAC*Δ*gdpP* bacteria were also analyzed by EM to uncover morphological defects caused by an increase in c-di-AMP. In contrast to the incomplete septa seen in cells with low c-di-AMP levels, *gdpP* mutant bacteria contained extra-membranous material at the division sites and in the form of vesicles in the cytoplasm (Fig. 3*C*). These data highlight that both high and low levels of c-di-AMP are detrimental to bacterial growth.

##### Global Changes in Gene Expression in Cells with Increased c-di-AMP Levels

**FIGURE 4.**
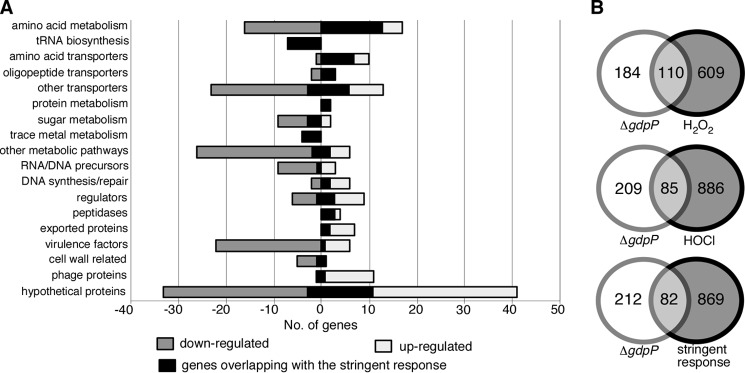
**Transcriptional changes in cells with high levels of c-di-AMP.** *A,* functional classifications of genes with altered transcript levels in strain LAC*Δ*gdpP* compared with wild type LAC*. The transcript levels of 307 genes were altered more than 2-fold in strain LAC*Δ*gdpP* (for full list see supplemental Table S1). These genes were grouped into different functional categories using KEGG mapper, and *light* and *dark gray bars* represent the 141 up- and 166 down-regulated genes, respectively. The *black shading* shows the 82 altered transcripts that match changes seen in *S. aureus* cells upon activation of the stringent response. *B,* Venn diagrams illustrating the overlap in transcriptional changes observed in the LAC*Δ*gdpP* with published microarray data sets. Of the 307 genes with altered transcripts, 294 were eligible for input into the SAMMD database. 37.4, 28.9, and 27.9% of these genes matched genes that were regulated in a similar manner in cells treated with H_2_O_2_, HOCl, or mupirocin to induce the stringent response, respectively.

Recently, several c-di-AMP receptor proteins have been identified in *S. aureus* (31), and these proteins are primarily associated with ion transport. To gain insight on a global level of the cellular pathways affected by an increase in c-di-AMP levels, microarray analysis was performed. RNA was extracted at the 4-h time point from the wild type LAC* bacteria (8.09 ± 0.96 μm c-di-AMP) and the LAC*Δ*gdpP* mutant strain (54.93 ± 8.15 μm c-di-AMP), and transcript levels were compared by whole genome microarray analysis. Using a 2-fold cutoff, 141 genes were up-regulated and 166 were down-regulated in the mutant compared with the wild type strain. As shown in Fig. 4*A* and supplemental Table S1, these 307 genes could be classified into different functional categories using the KEGG mapper. To determine whether this gene set overlaps with differentially regulated genes under other growth conditions, we made use of the *S. aureus* microarray meta-database (46), which contains data sets from other published microarray experiments performed on *S. aureus*. In total, 294 of the differentially expressed genes could be put into the database, and of these 37.4 or 28.9% matched gene expression changes observed in cells exposed to hydrogen peroxide for 30 min or hypochlorous acid for 10 min, respectively (Fig. 4*B*).

Hydrogen peroxide and hypochlorous acid are both reactive oxygen species (ROS) and a build up of ROS in the cell can damage DNA, RNA, proteins, and lipids. To limit the effects of ROS, bacteria contain multiple defense mechanisms, including DNA repair systems and enzymes such as superoxide dismutases to convert superoxides to hydrogen peroxide and oxygen. One of the transcripts altered upon deletion of *gdpP* is that of the superoxide dismutase SodM. When levels of c-di-AMP are high, there is a 5.56-fold down-regulation of *sodM*, indicating that these cells may not be able to effectively detoxify superoxide radicals produced during respiration. To examine whether the *gdpP* mutant is more susceptible to H_2_O_2_ stress or produces higher levels of peroxide, its sensitivity to H_2_O_2_ was determined using a disc diffusion assay, and peroxide levels were also measured during growth. However, no significant differences in the susceptibility to H_2_O_2_ or in peroxide levels were observed between the wild type and the *gdpP* mutant strains (data not shown). Therefore, it is as yet unclear why a number of altered transcripts in the Δ*gdpP* array match those of cells exposed to ROS. However, a significant number (27.9%) of genes differentially expressed under high c-di-AMP conditions also matched changes observed upon induction of the stringent response caused by the treatment of cells with 60 μg/ml mupirocin for 30 min (Fig. 4*A* and supplemental Table S1). Below, we present data showing that high levels of c-di-AMP trigger an RSH enzyme-dependent increase in (p)ppGpp production.

##### Stringent Response Alarmones (p)ppGpp Inhibit the Activity of GdpP

The stringent response is typically characterized by a down-regulation of genes involved in active growth, such as DNA replication and translation, and an up-regulation of genes involved in amino acid biosynthesis and stress resistance. Of the 82 genes altered in the Δ*gdpP* array and matching the stringent response, many are involved in amino acid metabolism, amino acid transport, and tRNA biosynthesis (supplemental Table S1 and Fig. 4*A*). During a previous study on *B. subtilis,* it was reported that the PDE activity of GdpP (named YybT in *B. subtilis*) is inhibited *in vitro* by the stringent response alarmone ppGpp (27). Together with the overlap in the transcript profile uncovered in this study, these results imply multiple interconnections between the c-di-AMP and stringent response signaling pathways.

**FIGURE 5.**
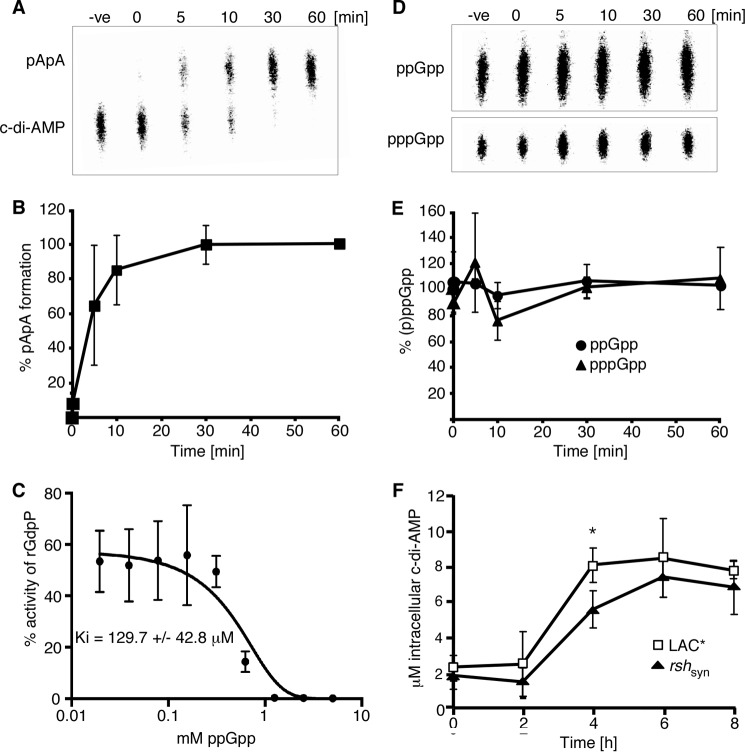
**Interplay between (p)ppGpp and c-di-AMP.** *A,* phosphodiesterase activity of rGdpP protein against c-di-AMP. Enzyme reactions were set up in assay buffer containing 1 μm rGdpP and 20 μm c-di-AMP. Reactions were stopped at the indicated time points and analyzed by TLC. *B,* quantification of phosphodiesterase activity of rGdpP against c-di-AMP. Enzyme reactions were set up as described in *A,* and pApA production was quantified. The % pApA formation was calculated, and the average value and standard deviations from three independent experiments were plotted. *C, in vitro* phosphodiesterase activity of GdpP in the presence of ppGpp. Enzyme reactions were set up with 1 μm rGdpP, 20 μm radiolabeled c-di-AMP and ppGpp at concentrations ranging from 0 to 5 mm. The hydrolysis of c-di-AMP to pApA was monitored by TLC, and the percentage of pApA reaction product was quantified using ImageQuantTL. The average values and standard deviations of three independent experiments were plotted, and the data were fitted using a dose response inhibition algorithm in GraphPad Prism with the corresponding *K_i_* value given. *D,* phosphodiesterase activity of rGdpP protein against (p)ppGpp. Enzyme reactions were set up in assay buffer containing 1 μm rGdpP and 20 μm (p)ppGpp. Reactions were stopped at the indicated time points and analyzed by TLC. *E,* quantification of phosphodiesterase activity of rGdpP against (p)ppGpp. Enzyme reactions were set up as described in *D* and ppGpp and pppGpp were quantified. The average value and standard deviations of the remaining amount of ppGpp and pppGpp in % from three independent experiments are plotted. *F,* intracellular c-di-AMP levels. The intracellular concentration of c-di-AMP in *S. aureus* LAC* and LAC*rsh_syn_ was determined as described in Fig. 1*C*.

To examine this in more detail, we first determined whether ppGpp could also inhibit the c-di-AMP hydrolase activity of the *S. aureus* PDE GdpP, making this a more general phenomenon and not specific to *B. subtilis* (27). For this, radiolabeled c-di-AMP was incubated with recombinant GdpP (rGdpP) protein lacking the N-terminal transmembrane helices, and the hydrolysis of the nucleotide to pApA was monitored by TLC. rGdpP was able to efficiently hydrolyze c-di-AMP in the absence of ppGpp (Fig. 5, *A* and *B*); however, this conversion was inhibited in a dose-dependent manner by increasing concentrations of ppGpp, and a *K_i_* of 129.7 ± 42.8 μm was determined (Fig. 5*C*). GdpP had no hydrolase activity against either ppGpp or pppGpp (Fig. 5, *D* and *E*), indicating that although these nucleotides inhibit the enzymatic function of this protein, they are not themselves substrates for GdpP.

Next, we examined whether the inhibition of GdpP by ppGpp has any effect on the levels of c-di-AMP in the cell. For this, we constructed an *rsh*_syn_ mutant strain, which, as described previously (35), is made defective in (p)ppGpp synthesis by removing three conserved amino acids in the (p)ppGpp synthesis domain of the RSH enzyme. We expect that in strain LAC*rsh_syn_, which is unable to synthesize (p)ppGpp, the PDE GdpP might be more active *in vivo*, potentially resulting in a decrease in intracellular c-di-AMP levels. To investigate this, cellular c-di-AMP levels were determined throughout the growth of the LAC*rsh_syn_ strain and the wild type LAC* control strain. This analysis revealed a trend toward reduced c-di-AMP levels in the *rsh*_syn_ mutant particularly at the 4-h time point (*p* = 0.0397), indicating that GdpP is more active in this strain (Fig. 5*F*). This suggests that in a wild type *S. aureus* strain undergoing nutrient starvation, high levels of (p)ppGpp may inhibit the activity of GdpP in the cell, resulting in higher levels of intracellular c-di-AMP.

**FIGURE 6.**
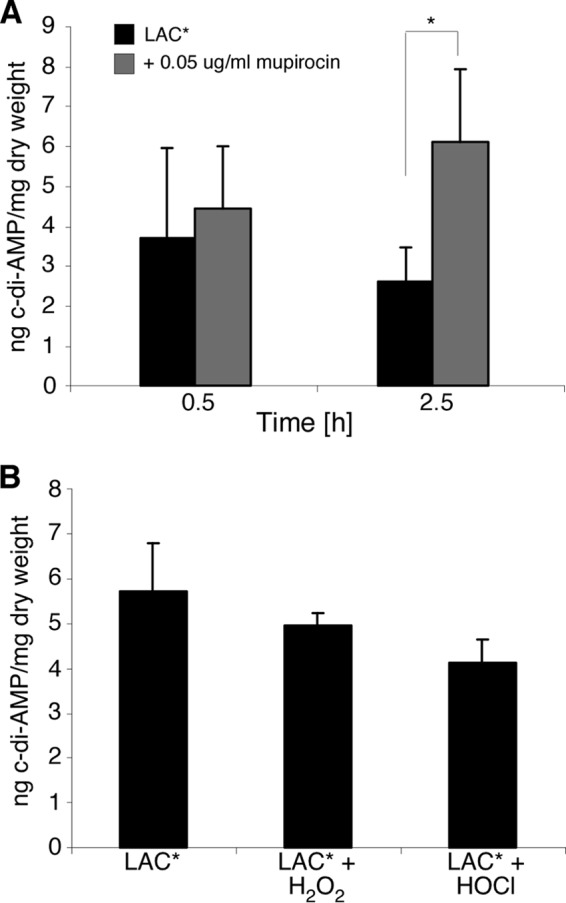
**c-di-AMP levels in cells exposed to stress.** *A,* levels of c-di-AMP in LAC* exposed to 0.05 μg/ml mupirocin were determined as described in Fig. 1*C*. LAC* was grown as described in Fig. 1*A,* and mupirocin was added at the 3.5-h time point. Cells were allowed to grow to the 4-h (0.5 h induction) and 6-h (2.5-h induction) time points before the extraction of cytoplasmic contents for analysis. *B,* intracellular concentration of c-di-AMP in *S. aureus* LAC* and LAC* exposed to 5 mm H_2_O_2_ or to 2.4 mm HOCl was determined as described above. LAC* was grown as described in Fig. 1*A*, with H_2_O_2_ added 30 min and HOCl added 10 min prior to the 4-h time point, at which time the cells were harvested for nucleotide extraction.

To analyze this, wild type LAC* was grown to mid-log phase, and the production of (p)ppGpp in the cell was induced with the addition of 0.05 μg/ml mupirocin. Induction of the stringent response led to an increase in the intracellular levels of c-di-AMP, particularly 2.5 h post-induction (*p* = 0.037) (Fig. 6*A*), indicating that (p)ppGpp might also inhibit the activity of GdpP *in vivo*.

##### High Levels of c-di-AMP Activate the Stringent Response through an RSH-dependent Increase in (p)ppGpp Production

*S. aureus* cells with high c-di-AMP levels show a significant transcriptional overlap with cells exposed to H_2_O_2_, HOCl, as well as with a stringent response transcriptional profile. Of the changes that occur however, only 6.5% are common to all three stimuli (supplemental Table S1), leading us to consider that all three responses are not mediated by a common mechanism and that the overlaps may not be c-di-AMP-dependent. To examine whether growth in the presence of H_2_O_2_ or HOCl leads to an increase in the production of c-di-AMP in the cell, and hence an overlap in transcription profile, we exposed cells to 5 mm H_2_O_2_ for 30 min or 2.4 mm HOCl for 10 min, conditions that replicate those used in the microarrays from the SAMMD database. This exposure however had no effect on the production of c-di-AMP (Fig. 6*B*).

**FIGURE 7.**
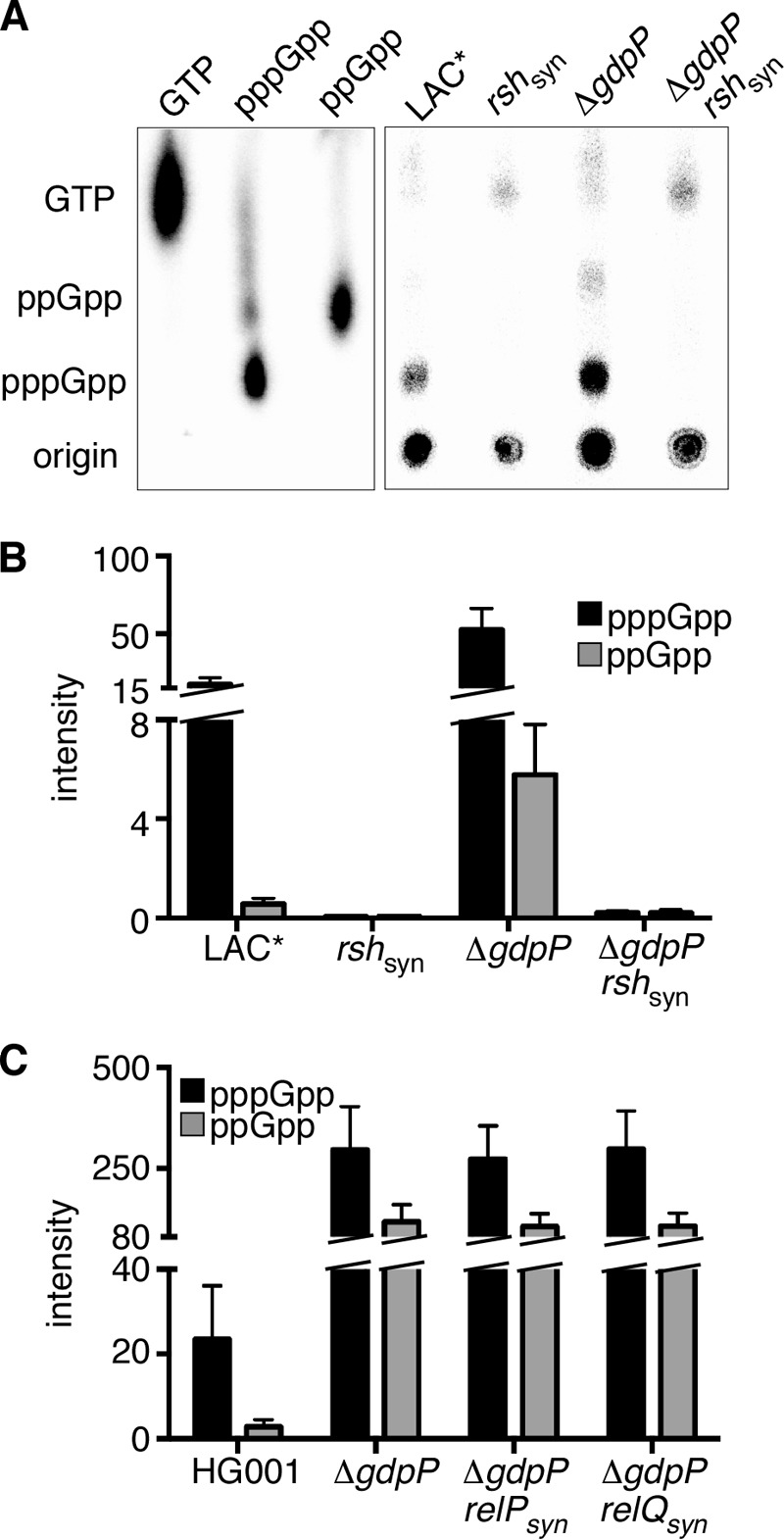
**Measurement of intracellular (p)ppGpp levels.** *A,* strains LAC*, LAC*Δ*gdpP,* LAC*rsh_syn_, and LAC*rsh_syn_Δ*gdpP* were grown in the presence of ^32^P-labeled H_3_PO_4_, and the stringent response was induced by the addition of mupirocin. The production of pppGpp and ppGpp was monitored by TLC, and radiolabeled GTP, pppGpp, and ppGpp (produced *in vitro*) were run in parallel to identify the relevant spots. *B,* quantification of intracellular (p)ppGpp levels. The radioactive spots corresponding to pppGpp and ppGpp from Fig. 7*A* were quantified using ImageQuantTL, and the data were plotted with GraphPad Prism. Average values and standard deviations from three independent experiments are shown. *C,* strains HG001, HG001Δ*gdpP,* HG001*relP*_syn_Δ*gdpP,* and HG001*relQ*_syn_Δ*gdpP* were grown in the presence of ^32^P-labeled H_3_PO_4_, and the stringent response was induced as described in *A*. The production of pppGpp and ppGpp was monitored by TLC, and the radioactive spots were quantified using ImageQuantTL. Average values and standard deviations from three independent experiments are shown.

Next, we wanted to determine whether c-di-AMP directly activates the genes associated with an increased stringent response or whether this overlap is caused by an increase in the production of the stringent response alarmones. To address this, ppGpp and pppGpp levels were measured in the wild type strain LAC*, the isogenic *gdpP* mutant, and the LAC*rsh_syn_ negative control strain. The three different strains were grown in LP-CDM in the presence of radiolabeled phosphoric acid, which acts as a phosphate donor for nucleotide synthesis. The stringent response was induced by the addition of mupirocin to culture aliquots, and the production of (p)ppGpp was examined by thin layer chromatography (TLC) (Fig. 7*A*). As expected, strain LAC*rsh_syn_ was unable to synthesize ppGpp or pppGpp even after the addition of mupirocin. Wild type LAC* produced moderate levels of both nucleotides, whereas the Δ*gdpP* strain produced 2.98 times (*p* = 0.013) higher levels of pppGpp and 10.22 times (*p* = 0.012) higher levels of ppGpp compared with the wild type strain (Fig. 7, *A* and *B*). This increase was also observed using the methicillin-susceptible *S. aureus* strain HG001, with a *gdpP* mutant containing 12.6 times higher levels of pppGpp (*p* = 0.012) and 41.4 times higher levels of ppGpp (*p* = 0.009) (Fig. 7*C*). By using (p)ppGpp synthase mutant strains, this activation of (p)ppGpp synthesis was shown to be dependent on the RSH enzyme (Fig. 7, *A* and *B*) but not RelP or RelQ, the two other synthases encoded on the *S. aureus* genome (Fig. 7*C*).

**FIGURE 8.**
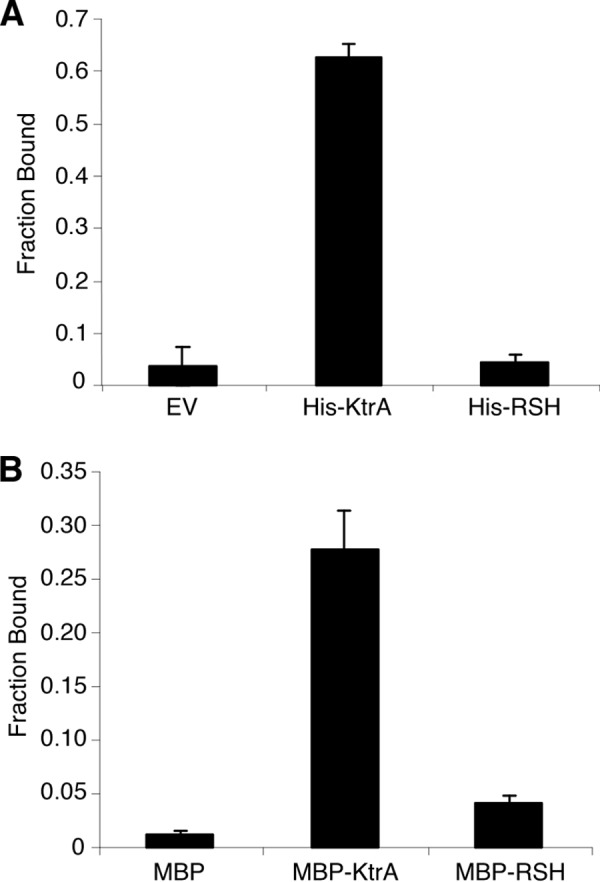
**Affinity of c-di-AMP for RSH.** Whole cell lysates of *E. coli* cells containing an empty vector (*EV*) or expressing either His-tagged KtrA or His-RSH (*A*) and purified MBP, MBP-KtrA, and MBP-RSH proteins (40 μm) (*B*) were mixed with ^32^P-labeled c-di-AMP in the presence of an excess of cold ATP, to ensure specificity, and c-di-AMP binding was examined. Fraction bound values were determined as described previously (39). Binding assays were performed twice. Averages and standard deviations are plotted.

Analysis of the Δ*gdpP* microarray data indicates that transcription of RSH is not increased in cells with high levels of c-di-AMP. To determine whether c-di-AMP can therefore directly bind to and activate the synthase activity of RSH, binding assays were performed using whole cell lysates expressing His-tagged RSH and radiolabeled c-di-AMP. Although c-di-AMP was able to bind to the positive control lysates expressing His-KtrA, a previously identified c-di-AMP-binding protein (31), no interaction was observed with either the whole cell lysates containing the empty vector or cells expressing His-RSH (Fig. 8*A*). To confirm the lack of binding, the assay was repeated using purified MBP-tagged RSH protein, again indicating that RSH and c-di-AMP do not interact directly (Fig. 8*B*). Taken together, these observations show that high levels of c-di-AMP lead to an activation of the RSH enzyme and an increase in (p)ppGpp levels. This activation is not achieved through a direct interaction between c-di-AMP and RSH, but rather through an indirect mechanism revealing a novel interconnection between the c-di-AMP signaling system and the stringent response.

## DISCUSSION

**FIGURE 9.**
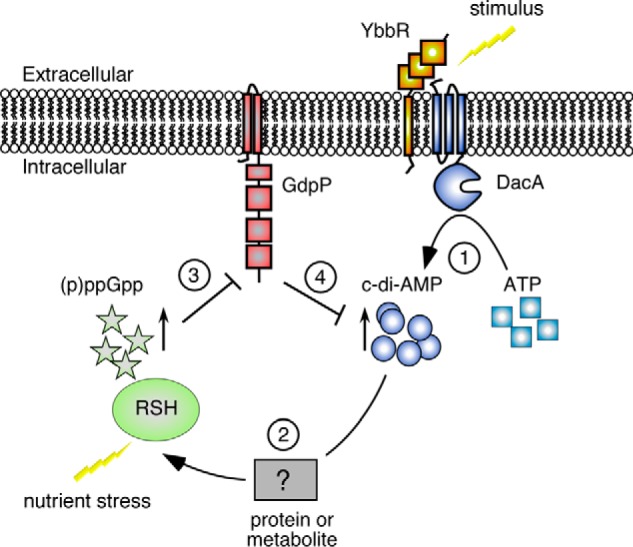
**Model depicting the interplay between the c-di-AMP and the stringent response signaling pathways.** As shown in this study, cellular c-di-AMP levels increase in *S. aureus* upon entry into stationary phase and likely also through other yet to be identified environmental signals. Stimuli could be sensed directly by the c-di-AMP cyclase DacA or by the membrane protein YbbR, which is encoded in the same operon. High cellular c-di-AMP levels (*step 1*) will, as shown in this work, also lead to an increase in intracellular (p)ppGpp levels (*step 2*) and activation of a subset of stringent response genes. Under nutrient limiting conditions, the intracellular (p)ppGpp concentration will rise to millimolar levels and inhibit the activity of the phosphodiesterase GdpP (*step 3*) leading in turn to an increase in c-di-AMP levels (*step 4*).

The different nucleotide signaling molecules and networks are classically viewed as distinct entities, as the nucleotides are produced and degraded by unique enzymes, whereas the target proteins usually respond with exquisite specificity to their corresponding nucleotides. However, cross-talk between different nucleotide signaling systems has emerged in more recent work. For example, a link between cGMP and cyclic di-guanosine monophosphate (c-di-GMP) signaling was recently demonstrated in the plant pathogen *Xanthomonas campestris,* where the production of cGMP enhanced the activity of a c-di-GMP cyclase, affecting virulence and biofilm formation (47). Additionally cAMP receptor protein, the cAMP-dependent transcription factor in *Vibrio cholerae,* has been shown to regulate the expression of the diguanylate cyclase CdgA, which plays a role in mediating biofilm formation (48). This study adds another layer of complexity to nucleotide signaling pathways in bacteria, as we show that the c-di-AMP and the stringent response networks are interconnected at multiple points in *S. aureus* (Fig. 9), and we would speculate that this is likely also the case in other Gram-positive bacteria. We show here that c-di-AMP levels increase when *S. aureus* enters stationary phase. The cellular changes triggering DacA cyclase and c-di-AMP production at this transition are still elusive. Based on the observation that different bacteria with mutations in *gdpP,* and therefore high levels of c-di-AMP, show increased resistance to acid, heat, and β-lactam antibiotics, this may indicate that such stresses might activate the system. However, it should be noted that direct evidence is still lacking that any of the above-mentioned stressors do indeed activate c-di-AMP production. As the nature of the stimuli is still unknown, one can only speculate as to how extracellular or intracellular changes are sensed. We would hypothesize that the sensor is either the c-di-AMP cyclase DacA itself or perhaps more likely the membrane protein YbbR, which is encoded in the same operon and co-occurs with *dacA.* Indeed in some bacteria a DacA-YbbR fusion protein seems to be produced (Fig. 9, *step 1*) (19). Furthermore, YbbR from *B. subtilis* has been shown to interact and increase the synthase activity of the c-di-AMP synthase DacA when expressed in a heterologous host (21). As shown in this study, at a high cellular c-di-AMP concentration the production of the stringent response molecules (p)ppGpp is promoted (Fig. 9, *step 2*), which we suggest would explain the observed overlap with the stringent response transcription profile. The c-di-AMP-induced alarmone production is achieved through activation of the RSH enzyme, but in an indirect manner as c-di-AMP does not bind to this enzyme. Additionally, the transcription of RSH is not altered in cells with high levels of c-di-AMP suggesting that the regulation of the production of this enzyme remains unchanged. To date, the only identified signal that can induce activity of RSH is amino acid deprivation (35). Although the known target proteins for c-di-AMP in *S. aureus* mostly include ion transporters (31), this molecule has been shown to negatively affect the *de novo* synthesis of the amino acid glutamate in *L. monocytogenes* (49). Although it is not thought that c-di-AMP has a role in the same pathway in *S. aureus* (49), it would still be of interest to examine any changes in amino acid levels in strains of *S. aureus* with increased c-di-AMP levels to determine whether this is the cause of the increased RSH activity.

This is, however, not the only connection between the stringent response and c-di-AMP signaling networks. As reported previously for *B. subtilis* (27), we show here that it is likely a more general phenomenon in Gram-positive bacteria that high levels of ppGpp can prevent the degradation of c-di-AMP by inhibiting the phosphodiesterase GdpP (Fig. 9, *step 3*). Therefore, activation of the stringent response, which in the case of *E. coli* results in the concentration of (p)ppGpp increasing to millimolar levels (3, 50, 51), will likely also cause an increase in c-di-AMP levels (Fig. 9, *step 4*). This is supported by the observation of a slight reduction in the cellular c-di-AMP levels in an *S. aureus* strain inactivated for the main ppGpp synthase RSH, as well as an increase in c-di-AMP in strains with higher levels of (p)ppGpp.

Our data have also highlighted that both high and low levels of c-di-AMP are detrimental to bacterial growth. This is in line with observations from *B. subtilis* (21, 24), *L. monocytogenes* (23), and *S. pneumoniae* (52) that show that depletion of the c-di-AMP cyclases in these organisms results in growth defects that are dependent on the loss of c-di-AMP. High levels of c-di-AMP also resulted in growth defects in both *B. subtilis* (21) and *S. pneumoniae* (52), with the *Bacillus* cells forming nonseparated curling filaments. Additionally, both high and low levels of intracellular c-di-AMP have been shown to be detrimental for bacterial survival *in vivo*, as deleting both PDE genes in *S. pneumoniae* attenuates bacterial virulence in a mouse pneumonia model of infection (52), although low levels of c-di-AMP attenuate the virulence of *L. monocytogenes* in both bone marrow-derived macrophages and in a mouse model (23). Whether these observed growth defects are related to altered potassium uptake or intracellular amino acid concentrations at deferring levels of c-di-AMP remains unclear. Taken together, our data on *S. aureus* and previous reports on other Gram-positive bacteria highlight that the synthesis of c-di-AMP must be tightly regulated as the intracellular levels of this secondary messenger are crucial for normal bacterial growth.

The microarray analysis performed in this study on *S. aureus* revealed that a total of 307 transcripts were altered in cells with high levels of c-di-AMP. This is in contrast to an array performed on a c-di-AMP PDE mutant in *L. monocytogenes* that identified 41 altered transcripts (23). Of the 41 genes altered on the *L. monocytogenes* array, only 14 are present in *S. aureus,* and excluding *gdpP,* which is due to its deletion, only the 2-fold up-regulation of the autolysin *atl* matched changes from the *S. aureus* array. Interestingly, when the PDE in *L. monocytogenes* was deleted, only a slight increase in c-di-AMP was observed in the culture supernatant (23). This is reminiscent of the situation in *S. pneumoniae,* where two PDEs, Pde1 (GdpP) and Pde2, are produced (52). Deletion of either one of these PDEs results in only a slight increase in c-di-AMP levels; however, deletion of both enzymes resulted in a 4-fold increase in nucleotide levels (52). The genome of *L. monocytogenes* also contains a gene encoding for Pde2, and it may be necessary for both of these PDEs to be deleted in *L. monocytogenes* to see the same increase in c-di-AMP level as in a *gdpP* mutant in *S. aureus*. Interestingly, the genome of *S. aureus* also contains a gene encoding for Pde2; however, due to the large increase in c-di-AMP levels in cells lacking GdpP (6.79-fold), we speculate that this PDE may not have a major role in c-di-AMP hydrolysis in *S. aureus,* at least not under standard laboratory growth conditions. Of the changes on the *S. aureus* array, no alterations in transcript levels for genes involved in fatty acid or lipid synthesis were observed. Therefore, the reason for the extra-membranous material seen in the EM images of the *gdpP* mutant strain remains unclear. Previously, we reported that high levels of c-di-AMP resulted in strains with increased peptidoglycan cross-linking and increased resistance to cell wall-targeting antimicrobials (22). One potential reason for this could have been the 6.17-fold increase in the transcription levels of the extracytoplasmic transcription factor SigS (supplemental Table S1), deletions of which have been linked to increased susceptibility to ampicillin and penicillin G (53). However, the introduction of a *sigS* deletion into the *gdpP* mutant strain did not alter the resistance profile, indicating that the increased resistance caused by high levels of c-di-AMP is SigS independent (data not shown). Of note, however, is a 2.71-fold increase in *pbp4* transcript levels in cells with high levels of c-di-AMP (supplemental Table S1). PBP4 is a low molecular weight penicillin-binding protein with transpeptidase activity. *pbp4* deletion mutants have been shown to have significant reduction in cross-linked peptidoglycan muropeptide (54, 55), and it is plausible that the increase in PBP4 in the *gdpP* mutant may explain this increased methicillin resistance phenotype.

Taken together, our data extend our knowledge of c-di-AMP function and turnover in *S. aureus*. We highlight the need for a tight control of cellular c-di-AMP levels to allow for optimal growth; but most importantly, we provide insights into a multilevel cross-talk between two important nucleotide-signaling molecules in bacteria.

## Footnotes

3 The abbreviations used are:

ppGpp: guanosine tetraphosphate
pppGpp: pentaphosphate
c-di-AMP: cyclic diadenosine monophosphate
PDE: phosphodiesterase
Atet: anhydrotetracycline
TEM: transmission electron microscopy
LP-CDM: low phosphate chemically defined medium
Erm: erythromycin
Cam: chloramphenicol
ROS: reactive oxygen species
MBP: maltose-binding protein.
